# Sellar xanthogranuloma as a diagnostic challenge: a report on five cases

**DOI:** 10.3389/fnins.2023.1227144

**Published:** 2023-09-22

**Authors:** Silvia Carolina Fernández, María Celina Bernhardt, Ezequiel Grondona, Ana Clara Venier, María Lorena Bertolino, Mauro José Pautasso, Emilio Mezzano, Roxana Analía Damilano, Claudia Susana Sala, Enrique José Herrera, Favio Nicolás Pesaola, Cristina Alicia Maldonado, Amado Alfredo Quintar, Ana Lucía De Paul

**Affiliations:** ^1^Centro de Microscopía Electrónica, Facultad de Ciencias Médicas, Universidad Nacional de Córdoba, Córdoba, Argentina; ^2^Servicio de Patología, Clínica Universitaria Reina Fabiola, Córdoba, Argentina; ^3^Instituto de Investigación en Ciencias de la Salud (INICSA), Consejo Nacional de Investigaciones Científicas y Técnicas (CONICET), Córdoba, Argentina; ^4^Servicio de Neurocirugía, Clínica Universitaria Reina Fabiola, Córdoba, Argentina; ^5^Servicio de Endocrinología, Clínica Universitaria Reina Fabiola, Córdoba, Argentina; ^6^Servicio de Endocrinología, Sanatorio Allende, Córdoba, Argentina; ^7^Servicio de Neurocirugía, Sanatorio Allende, Córdoba, Argentina; ^8^Department of Pediatrics, School of Medicine, Genetics and Genomic Medicine, Washington University in St. Louis, Saint Louis, MO, United States

**Keywords:** xanthogranuloma, chronic inflammation, sellar region, Rathke’s cleft cyst, pituitary adenoma, MRI, electron microscopy

## Abstract

Xanthogranulomas are considered rare tumors, with their sellar and non-sellar frequency ranging from 1.6 to 7% among intracranial lesions, and described as a separate entity by the World Health Organization in 2000. The diagnosis of sellar xanthogranulomas is challenging, given their uncertain origin and clinical course. In addition, the limited reporting of sellar xanthogranuloma cases and the absence of characteristic images make these entities difficult to distinguish from other cystic lesions of the sellar region, such as adamantinomatous craniopharyngiomas, Rathke’s cleft cysts, pituitary tumors, arachnoid cysts, epidermoid cysts, and dermoid cysts. Here, we describe the clinical presentation, radiological findings, immunohistochemical/histopathological analysis, and the ultrastructural examination by transmission electron microscopy of five sellar xanthogranulomas cases reported in two care centers in Cordoba, Argentina. Two males and three females between 37 and 73 years of age (average 51.8 years) presented with persistent headaches, generalized endocrine defects, and visual problems. MRI revealed cystic formations in the sellar region, which usually projected into adjacent tissues such as the suprasellar region or cavernous sinuses, and compressed other structures such as the optic chiasm, pituitary gland, and cranial nerves. All patients underwent surgical intervention to remove the tumor tissue. The histopathological analysis of the samples showed cellular tissue with a xanthogranulomatous appearance, inflammatory cellular infiltrate (mainly lymphocytes and macrophages), fibroblasts, abundant collagen fibers, and hemorrhages. An ultrastructural analysis helped to identify cellular infiltrates and granules resulting from tumor cell activity. The data support the hypothesis that sellar xanthogranulomas could occur as an inflammatory reaction secondary to the rupture and hemorrhage of a previous cystic process, thereby generating an expansion of the tumor body toward adjacent tissues. The information obtained from these cases contributes to the current knowledge about this disease’s origin and clinical and histological evolution. However, the scarcity of patients and the observed phenotypic heterogeneity make its diagnosis still challenging. Undoubtedly, more investigations are needed to provide additional information in order to be able to achieve a more accurate diagnosis and effective treatment of this rare disease.

## Introduction

Sellar xanthogranulomas (SX) are rare chronic inflammatory lesions secondary to hemorrhages, inflammation, infarction, and/or necrosis of a pre-existing Rathke’s cleft cyst, craniopharyngioma, or pituitary tumor (Lozovanu et al., 2022). The incidence of both sellar and non-sellar intracranial xanthogranulomas is 1.6–7%, which occurs mainly in the choroid plexus of the trigone of the lateral ventricles (Dai et al., 2017; La Rocca et al., 2019), and in much rarer cases in the sellar or parasellar region, with a prevalence of ~0.6% among pituitary tumors (Rahmani et al., 2015; Ved et al., 2018). The etiology and pathogenesis of SX are still unclear, with several conflicting theories having attempted to explain their heterogeneous origin. The most frequently postulated hypothesis has suggested that they arise as the final expression of a secondary reaction to another pre-existing lesion (Nishimura et al., 2018). However, it is still unclear whether SX are a distinct entity or a particular inflammatory response to which the different pathologies derive (Honegger et al., 2021).

Histopathological findings usually reveal cholesterol spikes, hemosiderin deposits, chronic inflammatory cell infiltrates, multinucleated giant cells, macrophages, and fibrous proliferation (Amano et al., 2013). In addition, small remains of squamous or cuboid epithelial tissue are often found, including tubules and glands, which, due to their cytoarchitectural features and immunohistochemical profile, cannot be linked to an adamantinomatous craniopharyngioma characterized by the nuclear accumulation of beta-catenin (Honegger et al., 2021).

Their clinical presentation is frequently associated with headaches (50% of cases), visual defects (63–67%), central diabetes insipidus (33–43%), and anterior pituitary dysfunction (67%; Amano et al., 2013; Hernández-Estrada et al., 2017; La Rocca et al., 2019). Moreover, they are difficult to differentiate from other sellar lesions in the pre-surgical stage due to the lack of characteristic biochemical biomarkers or imaging findings (Dai et al., 2017). They present a variable signal intensity on magnetic resonance imaging (MRI) which depends on the predominance of each histological component. For example, although cholesterol crystals are observed to be hyperintense on T1 and hypointense on T2, hemosiderin cysts are characterized by an iso- or hyperintense signal on T1 and hyperintense on T2, while fibrosis (granulation) appears hypointense on both T1 and T2. Furthermore, the enhancement is usually heterogeneous after contrast medium administration (Hernández-Estrada et al., 2017). Regarding the location of xanthogranulomas, they are most frequently found to be intrasellar with a suprasellar origin (67%), less regularly only suprasellar (22%), and are rarely exclusively intrasellar (11%; Burt et al., 2003; Nishimura et al., 2018; Louis et al., 2021).

The preferable treatment for xanthogranulomas is the surgical reduction and decompression of the optic chiasm with preservation of the pituitary stalk (Burt et al., 2003; Yang et al., 2017). However, considering the limited number of cases reported in the literature, recognizing and addressing this type of injury is still a challenge. This work describes five SX cases reported in two care centers in Cordoba, Argentina.

## Case 1

**Figure 1:**
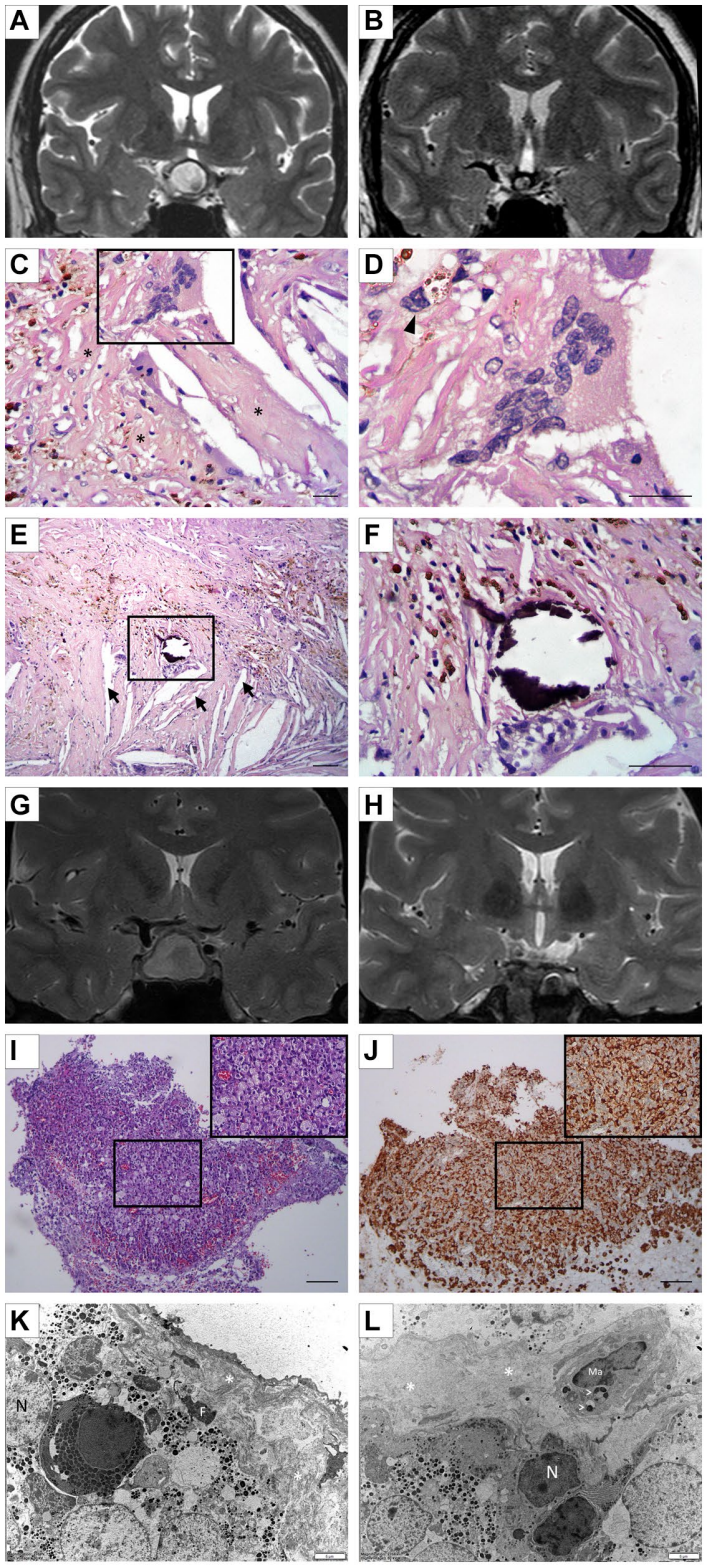
**(A)** Case 1: Pre-surgical coronal pituitary MRI showing a solid cystic tumor lesion (17 mm × 14 mm). A hyperintense cystic portion and a small hypointense left paramedian eccentric solid mural nodule can be seen on T2. **(B)** Case 1: Post-surgical coronal MRI exhibiting changes at the sellar and suprasellar level. Minimal hemorrhagic changes, lateralized to the left, without any clearly visible tumor lesion, are seen in T2. **(C,D)** Case 1: Representative image of XS showing multinucleated giant cells; hemosiderophages (arrow head); and reactive fibroblastic proliferation (*). Morphological details of multinucleated giant cells (**D**; H/E staining). Scale bar 20 μm. **(E,F)** Case 1: Microscopical examination of XG showing a solid area composed of a mixture of tumoral/inflammatory cells, and the focus of dystrophic microcalcification associated with clear acicular cholesterol crystals (arrow) immersed in an inflammatory process **(E)**. **(F)** Details of microcalcification (H/E staining). Scale bar 20 μm. **(G)** Case 2: Preoperative coronal MRI of sellar lesion measuring 12 mm × 17 mm × 20 mm, faintly hyperintense on T2 with hypointense margins. The lesion is compressing and displacing the chiasm and optic pathways. **(H)** Case 2: Postoperative coronal MRI revealing a 4 mm remnant in the central sector, hypointense on T2 coronal, and indicative of hemosiderin deposit. Areas in the periphery are suggestive of glandular parenchyma. **(I,J)** Case 2: Representative images of histopathological and immunohistochemical staining of resected tumoral tissue. Diffuse infiltration of macrophage mononuclear cells with foamy cytoplasm with a xanthomized appearance are seen (**I**; H/E staining). A strong and diffuse immunolabeling for CD68 is clearly detected in macrophages (**J**; 3, 3′-diaminobenzidine staining). Scale Bar 100 μm. **(K)** Case 2: Representative electron micrographs of pituitary tissue depicting numerous epithelial cells containing several secretory granules of different sizes. Next to these cells, the presence of dense connective tissue (*) with a fibrocyte **(F)** is highlighted. N, nuclei; F, fibrocyte. **(L)** Case 2: A macrophage (Ma) is immersed in the pituitary parenchyma, exhibiting phagocytic vacuoles (arrowhead) probably containing secretory granules in the process of digestion. N, nuclei; Ma, Macrophage; dense connective tissue (*).

A 44-year-old female patient with a 1-year history of neck pain accompanied by headache. Cervical spine studies incidentally showed a tumor in the sellar-suprasellar region. A pituitary MRI revealed an expansive heterogeneous cystic, solid tumor lesion of 17 mm × 14 mm with regular contours. The cystic portion of the lesion showed high signal intensity on both T1 and T2 sequences. Additionally, a small eccentric left paramedian solid mural nodule of 8 mm × 5 mm is observed, which appears hypointense on T2. The tumor extends suprasellar and compresses the pituitary stalk and optic chiasm (Figure 1A). The pre-surgical assessment of the pituitary function was normal. A campimetric study of the right eye revealed a narrowing of the nasal perimeter, while the left eye exposed an incomplete, inferior temporal quadrantopia. Surgery was performed transcranially. The histopathological findings were compatible with xanthogranulomas: the sample was entirely composed of dense connective tissue, with sectors of fibrosis and chronic inflammatory reaction with lymphocytes and frequent macrophages, some of which exhibited a xanthomized appearance associated with multinucleated giant cells (Figures 1C,D). Cholesterol spikes, the foci of dystrophic microcalcification, and signs of an old bleeding (hemosiderin-filled hemosiderophages) were also detected (Figures 1E,F). A post-surgical MRI showed modifications at the sellar level with a residual thinning of the adenohypophysis, whose remnants were attached to the sellar floor, and with small hemosiderin deposits in its left lateral recess. In addition, the pituitary stalk was deviated to the right and slightly thickened (Figure 1B).

## Case 2

A 37-year-old female patient consulted for evaluation of postpartum secondary amenorrhea and galactorrhea. She had been taking antidepressants for 3 months. In addition, the patient manifested polyuria and polydipsia. The hormonal evaluation showed hypogonadotrophic hypogonadism and hyperprolactinemia (PRL: 248.2 ng/mL). The thyroid function and androgen levels were normal. Cabergoline, 0.5 mg per week, was indicated. A pituitary MRI revealed a heterogeneous lesion at the adenohypophysis level measuring 12 mm × 17 × 20 mm, which was slightly hyperintense and with hypointense margins on T2 and isointense signal on T1, without any enhancement after intravenous contrast (Figure 1G). The lesion was compressing and displacing the optic chiasm and cephalic tracts. Surgery was performed *via* the transseptosphenoidal approach with an endonasal approach. The histopathological analysis showed an extensive xanthogranulomatous chronic inflammatory reaction composed of abundant clumped foamy macrophages (Figure 1I), some multinucleated giant cells, and lymphocytes. Scarce pituitary tissue with a preserved hormonal histoarchitecture was also observed. The immunohistochemical staining revealed strong and diffuse CD68-positive macrophage staining (Figure 1J). At the electron microscopy level, it was observed that the endocrine cells were intermingled with fibroblasts, abundant collagen fibers, and inflammatory cells (mainly macrophages; Figures 1K,L). The post-surgical MRI showed alterations of surgical origin at the sellar level, with focal dehiscence of the sellar floor and a 4-mm remnant in its central sector. In addition, areas of homogeneous enhancement were observed in the periphery after intravenous contrast administration, suggesting remnants of a glandular parenchyma. Neurohypophysis was not identified (Figure 1H).

## Case 3

**Figure 2:**
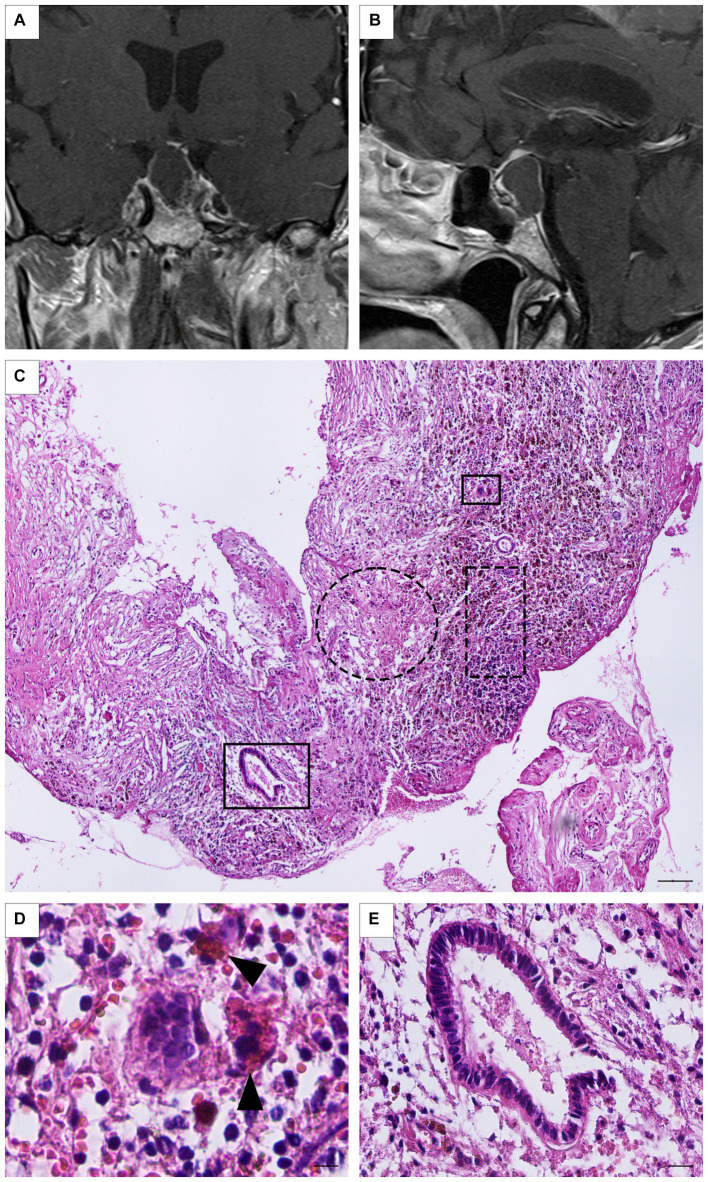
**(A,B)** Case 3: Preoperative coronal and sagittal MRI showing a large, thick-walled, cystic-like lesion (28 mm × 19 mm × 19 mm), hypointense on T1, extended to the suprasellar cistern. Neither the signal corresponding to the neurohypophysis nor the pituitary stalk are identified. **(C)** Panoramic photomicrograph showing dense connective tissue (dotted circle) and frequent macrophages (dot rectangle; H/E staining). Scale Bar 100 μm. **(D)** Multinucleated giant cell and hemosiderin-laden macrophages can be detected (arrow head; H/E staining). Scale bar 20 μm. **(E)** Simple columnar tubular epithelial rest immersed in an inflammatory background (H/E staining). Scale bar 20 μm.

A 63-year-old female patient consulted due to intense persistent headaches for 15 days, located retro-orbitally, which did not respond to analgesics, and only partially responded to amitriptyline. The patient did not manifest any other relevant symptoms. Her personal history included arterial hypertension and dyslipidemia. The clinical laboratory did not show any alterations in the pituitary hormonal axes, and the computerized visual field was defect-free. A brain MRI exposed a spontaneously hyperintense image in the sellar region with a suprasellar extension, which suggested a craniopharyngioma or Rathke’s cleft cyst. The patient returned to control with the endocrinologist 6 years later with persisting chronic, left-sided, and retro-orbital headaches. In addition, she had noted a decrease in her peripheral vision in the months before the consultation. A pituitary MRI showed a voluminous lesion of 28 mm × 19 mm × 19 mm, hypointense on T1 and hyperintense on T2, with a thick-walled cystic appearance that occupied the entire *sellae turcica* and extended to the suprasellar cistern, compressing and displacing the optic chiasm. It also caused lysis and remodeling of the sphenoid bone in anterior and posterior directions while compressing both cavernous sinuses in a lateral direction (Figures 2A,B). The pituitary profile revealed no hormonal deficits, and the computerized visual field was normal. Surgery was performed by a transsphenoidal endoscopic approach. A histopathological examination revealed an extensive chronic xanthogranulomatous-type inflammatory reaction, represented by abundant foamy macrophages interspersed with multinucleated giant cells, lymphocytes, and neutrophils associated with cholesterol spikes (Figures 2C,D). In addition, signs of recent bleeding and isolated tubular epithelial structures were observed lined by cylindrical epithelium, without atypia, which were closely related to the inflammatory process (Figures 2C-E). Nine months after surgery, a new sellar image exposed a cystic lesion at the clivus level, suggesting recurrence, which motivated a new surgical intervention *via* a transsphenoidal approach. The anatomopathological study confirmed the diagnosis of SX, similar to that that reported after the first surgical intervention. Three months after the second surgical intervention, the sellar MRI showed no changes compared to the previous study of the cystic lesion. Thus, radiosurgery was indicated (IMRT 5 fractions, total dose of 35 Gy). To date, the patient remains clinically asymptomatic, with no evidence of a deficit in the visual field and preserved pituitary axes, and is under expectant management.

## Case 4

**Figure 3:**
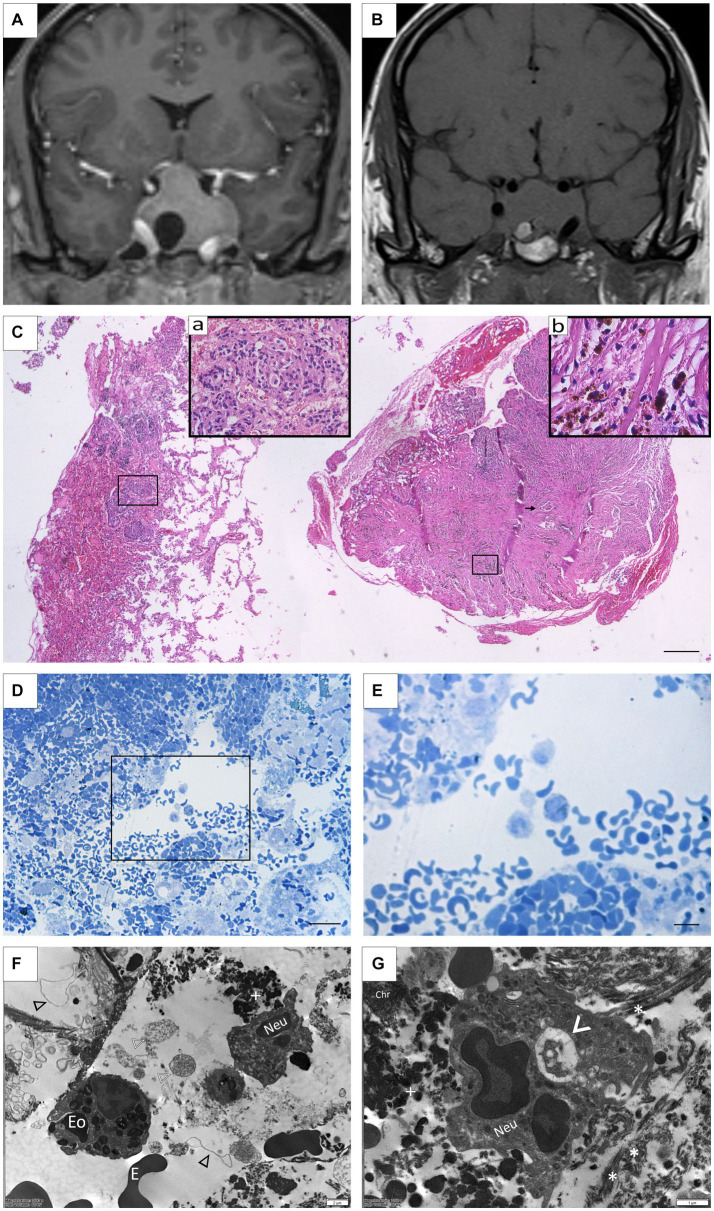
**(A)** Case 4: Pre-surgical coronal MRI showing a solid expansive lesion measuring 39 mm × 31 mm × 32 mm, hypertense on T1 with gadolinium. An eccentric area with a cystic appearance in the right lateral sector is also seen. **(B)** Post-surgical coronal MRI showing an intrasellar lesion measuring 25 mm× 35 mm× 27 mm with suprasellar growth infiltrating both carvenous sinuses, isointense on T1. An area of intratumoral hemorrhage in the right lateral basal is also detected. **(C)** Panoramic photomicrograph of surgical specimen revealing leftward neuroendocrine pituitary tumor with signs of ischemia, interstitial hemorrhage, and erythrocyte extravasation. To the right, dense fibroconnective tissue with slits, abundant hemosiderophages, and cell multinucleated giants (arrow). (Insert **a**) Pituitary neuroendocrine tumor composed of chromophobe cells with interstitial hemorrhage and signs of ischemia. (Insert **b**) Hemosiderophages and macrophages. (H/E staining). Scale Bar 100 μm. **(D,E)** Remnants of tumoral tissue showing extensive hemorrhage and macrophage activation (Toluidine blue staining). Scale Bar 100 μm **(D)**; 20 μm **(E)**. **(F)** Polimorfonuclear immune cells, including eosinophils (Eo), neutrophils (Neu), and erythrocytes (E) are frequently observed as part of the connective tissue forming the sellar xanthogranulomas. In the background, detritus from necrotic cells, including membranes (black arrowhead), nuclei fragments (white arrowhead), and organelles (white cross) can also be seen. **(G)** At higher magnification, a neutrophil (Neu) is observed engulfing debris material from the microenvironment (arrowhead), which is comprised of collagen fibers (*), granular remains (white cross), and free chromatin (Chr).

A 42-year-old male patient attended consultation due to 2 months of a persistent left temporoparietal headache without any visual alterations or relevant clinical history. A sellar MRI revealed an expansive intrasellar lesion with suprasellar growth toward both cavernous sinuses of 39 mm × 31 mm × 32 mm, which compressed the optic chiasm and the prechiasmatic portion of both optic nerves. It appeared as a solid lesion with an eccentric area, having a cystic appearance in the right lateral sector, and a hypointense signal on T1. The lesion included the left internal carotid in its cavernous course, the third, fourth, and sixth cranial nerves, and the V1-V2 segments of the trigeminal (Figures 3A,B). The campimetry evaluation was normal, but a hormonal evaluation revealed panhypopituitarism with involvement of the thyroid, adrenal, and gonadal axis. Hydrocortisone 20 mg/day and levothyroxine 100 mcg/day were indicated, and the patient underwent surgery via the transseptosphenoidal approach. The histopathological findings were compatible with a pituitary tumor. A post-surgical MRI reported an expansive sellar injury with intralesional hemorrhage, suggesting a persistent tumor. The patient was again subjected to surgery by a transseptosphenoidal approach 6 months later. New histopathological findings showed a neoplastic proliferation of epithelial cells corresponding to a pituitary neuroendocrine tumor. A small fragment with fibroblastic proliferation, collagen fiber deposition, mononuclear infiltrate of lymphocytes, some hemosiderophages, and occasional foreign body-type multinucleated giant cells (secondary inflammatory reaction) was detected (Figure 3C). Residual neoplastic epithelial tissue with signs of active bleeding and evidence of macrophage activation was also observed (Figures 3D,E). Abundant inflammatory cells (mainly eosinophils and neutrophils) appeared surrounded by a great deal of cellular detritus, such as membranes, apoptotic bodies, and necrotic cells, together with blood extravasation as revealed by electron microscopy (Figure 3F). Moreover, neutrophils exhibited intense phagocytic activity (Figure 3G). Finally, immunohistochemical assays revealed diffuse FSH-positive staining with variable intensity, focal and weak LH staining, and a Ki67 index of 4% of neoplastic cells.

## Case 5

**Figure 4:**
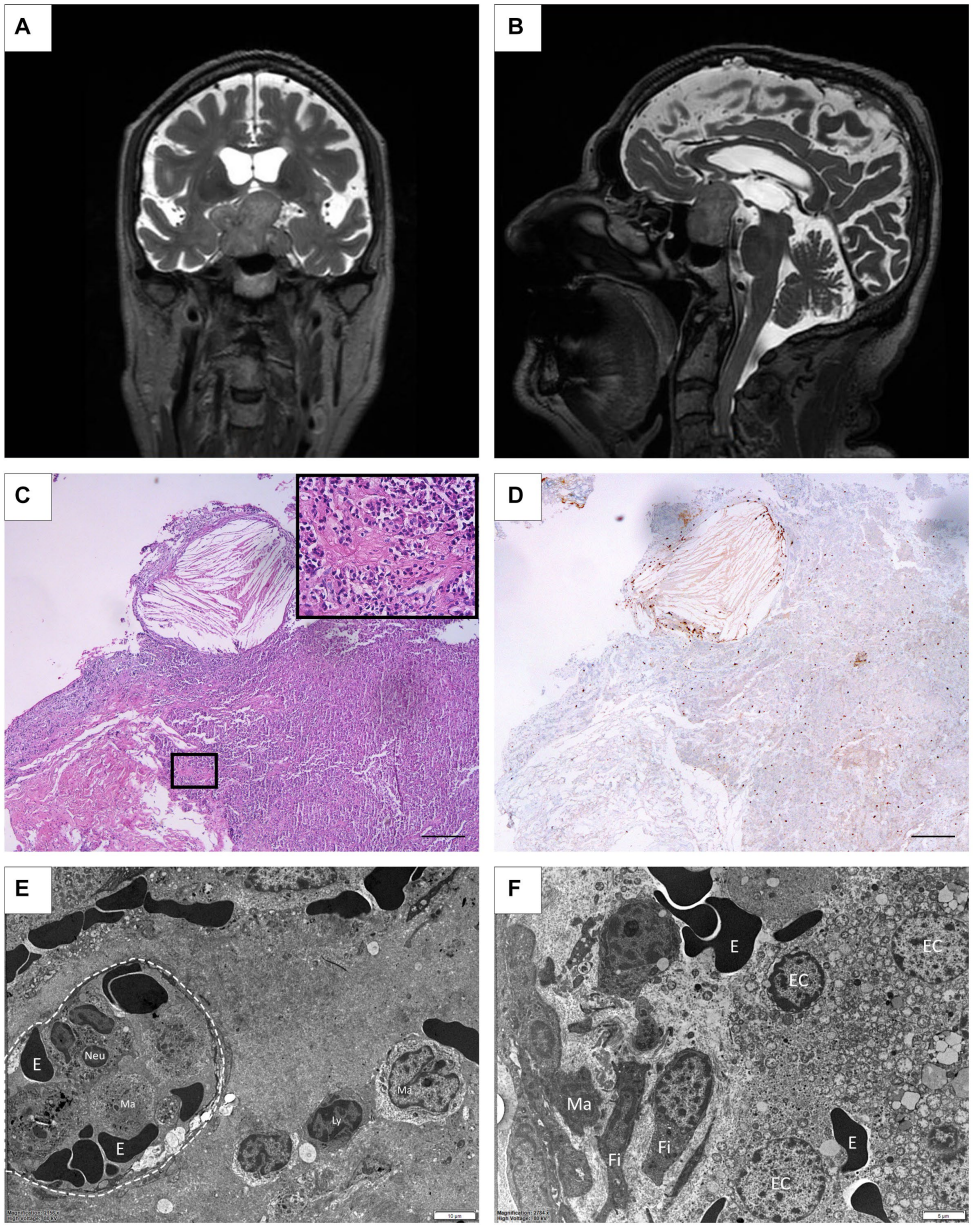
**(A,B)** Case 5: Pre-surgical MRI coronal and sagittal showing a solid, polylobulated lesion, 35 mm × 27 mm × 38 mm, hyperintense on T2 with some cystic areas. An extension toward the suprasellar cistern, generating a mass effect on the hypothalamus and the optic chiasm is shown invading both cavernous sinuses. **(C)** Histopathological examination showing xanthogranulomatous inflammatory reaction with abundant cholesterol spikes immersed in the pituitary neuroendocrine tumor. Inset: Chromophobe: monomorphic pituitary tumor cells distributed in a solid pattern are shown. (HE staining) Scale Bar 100 μm. **(D)** Nuclear immunohistochemical staining of Ki67 in neoplastic cells. (3, 3′-diaminobenzidine staining). Scale Bar 100 μm. **(E)** Electron microscopy picture depicting a capillary structure (dotted circle) containing erythrocytes (E) and inflammatory cells in the lumen. Lymphocytes (Ly) and mononuclear monocytes/macrophages (Ma) are also seen in the surrounding tissue. **(F)** Microphotograph showing epithelial secretory cells (EC) alongside extravasated erythrocytes (E). In addition, the connective tissue displays fibroblasts (Fi) and infiltrated macrophages (Ma).

A 73-year-old male patient attended consultation with a history of colon cancer, ischemic heart disease, and bitemporal hemianopsia, with the latter being confirmed by the ophthalmology service after an episode of dizziness. A sellar MRI showed a solid, polylobed, space-occupying formation of 35 mm × 27 mm × 38 mm, which was visualized as isointense on the T1 sequence, heterogeneous on the T2 sequence, and predominantly hyperintense with some cystic areas. The lesion presented some hyperintense foci, which may correspond to hemosiderin or calcifications. It extended cephalad, obliterating the suprasellar cistern, generating a mass effect on the hypothalamus and the optic chiasm, displacing both optic nerves, invading both cavernous sinuses, and surrounding the internal carotid arteries (Figures 4A,B). A hormonal evaluation showed panhypopituitarism with involvement of the thyroid, adrenal, and gonadal axis. Levothyroxine 88 mcg/day, hydrocortisone 15 mg/day, and testosterone enanthate 250 mg every 28 days were indicated, and the patient underwent surgery via a transseptosphenoidal with an endonasal approach. Histological sections showed an epithelial neoplastic cell proliferation of cuboidal to low cylindrical morphology, of medium size and generally monomorphic, and associated with a pituitary neuroendocrine tumor. Signs of hemorrhage and the foci of an intratumoral xanthogranulomatous reaction were also recognized (Figure 4C). A tumoral infiltration toward a dense connective tissue (possibly meninges) was also observed in some fragments. Immunohistochemical assays revealed focal and moderate LH staining, focal and weak FSH labeling, and a Ki67 index of 5% of neoplastic cells (Figure 4D). At the electron microscopy level, several inflammatory cells, including monocytes, lymphocytes, and neutrophils, could be observed, together with fibroblasts and abundant collagen fibers (Figure 4E).

Active tumoral cells, with numerous mitochondria and small secretory granules aligned to the plasma membrane, and tumoral necrotic cells next to connective tissue, were also detected (Figure 4F).

## Discussion

The SX are sporadic intracranial lesions and only a few cases have been reported in the literature (Ved et al., 2018). In this work, we present five cases of this sellar entity diagnosed in two medical centers in Cordoba, Argentina.

Our case series was characterized by presenting a wide range of xanthomatous lesions, either isolated or associated with pituitary adenomas. The average age of presentation was 51 years, consistent with previous reports (Amano et al., 2013; Rahmani et al., 2015), and three out of five patients were female, supporting the slight female preponderance described elsewhere (Hernández-Estrada et al., 2017). The most frequent clinical presentation was headaches with endocrine dysfunction (three patients), in line with reported xanthogranulomas being linked to headaches or hypopituitarism (Amano et al., 2013; Hernández-Estrada et al., 2017).

In the present study, we have described the radiological findings, which were typical. However, imaging features may overlap with other sellar and suprasellar neoplasms, making it challenging to establish a definitive diagnosis based on imaging alone. In our case series and, in agreement with reports from Ved et al. (2018) and Lozovanu et al. (2022), the presence of total or partially cystic morphology (with a sellar or intrasellar-suprasellar location), hyperintensity on T1 and T2 sequences, and no evidence of cavernous sinus invasion, emerge as a combination of imaging features to be considered in the preoperative differential diagnosis of xanthogranuloma. Therefore, a comprehensive evaluation, including clinical, radiological, and histopathological correlation, is necessary to establish a definitive diagnosis. For radiologists, endocrinologists and clinicians, it is important to be aware of the possibility of xanthogranuloma and consider it in the differential diagnosis when encountering characteristic imaging features in sellar or suprasellar lesions.

Macroscopical features of SX include a fibrous capsule densely adherent to the dura, and a tumoral mass with non-hemorrhagic soft tissue and cystic components (Hernández-Estrada et al., 2017). This may arise as a result of a chronic inflammatory process, whose histopathological characteristics include the presence of infiltrated foamy macrophages, multinucleated giant cells, hemosiderin deposits, and evidence of central necrosis (Kleinschmidt-DeMasters et al., 2017). However, the histomorphological features may vary depending on the evolution time, with the cellular component predominating in early lesions and the fibroconnective element in evolved ones. Therefore, applying appropriate histopathological studies is of particular importance to rule out underlying pathologies, even more so when considering that xanthogranuloma is frequently a diagnosis of exclusion. In this sense, it is strongly recommended to process all the biopsy material obtained in the surgical act, as well as to access the information provided by images and the clinical and surgical semiology of the patient, to be able to achieve an adequate correlation.

In the initial description of SX, Paulus et al. (1999) described the presence of cholesterol clefts and lymphocytic infiltrates in 100% of cases, followed by hemosiderin deposits (indicative of past bleeding, 89%), fibrosis (86%), foreign body giant cells (78%), eosinophilic necrotic debris (59%), and macrophage accumulation (59%). Since histopathological analysis of these lesions is usually purely morphological, auxiliary histochemical and immunohistochemical techniques, such as the CD68 detection specific for macrophages, is strongly recommended when few tissue samples are available.

The contribution of electron microscopy is an aspect worth highlighting from this present study, which provided valuable information from ultrastructural details and exposed some of the etiological factors that trigger the aforementioned inflammatory process. This approach allowed the precise identification of ultrastructural features in both the proliferating epithelial compartment and immune infiltrating cells (De Paul et al., 2009; Sabatino et al., 2018; Scalerandi et al., 2018). After necrosis, interstitial hemorrhage and cell damage generate lipid-rich membrane remnants that favor the extracellular deposition of cholesterol and secondary crystal formation, thereby providing a chemotactic attractant for inflammatory cells, predominantly macrophagic mononuclear cells. For instance, the presence of eosinophils, histiocytes, and the whole spectrum of macrophage-like cells was confirmed by electron microscopy.

The pathogenesis of SX is controversial. The histopathological characteristics and the evident presence of chronic inflammatory cells have fueled the theory that SX are on the same spectrum as Rathke’s cleft cysts and craniopharyngiomas, representing a potential side reaction caused by repeated swelling and bleeding. In fact, it often originates with the rupture of a cyst, such as Rathke’s cleft cysts, leading to the recruitment of inflammatory cells and macrophage activation. This is accompanied by local tissue damage, lipid release, and cholesterol crystal formation, thus perpetuating the granulomatous response. Craniopharyngioma, usually the adamantinomatous subtype, is one of the neoplasms that can have degenerative changes, such as cystification, calcification, and xanthogranulomatous reaction (Müller et al., 2012; Louis et al., 2021). Similar modifications have also been recognized in pituitary neuroendocrine tumors, especially macroadenomas and giant adenomas, with secondary phenomena of bleeding or necrosis (Nishioka et al., 2010; Amano et al., 2013; Li et al., 2018; Vasquez et al., 2019). The presence of xanthogranulomatous findings in these tumors was incidental and should not directly influence the natural course or behavior of the neoplasm.

On the other hand, SX are not specific to the sellar region, which motivated previous classifications to include them into the category of “xanthomatous reactions of the nervous system,” alongside other conditions of metabolic, degenerative, and reactive origins, which are usually non-neoplastic pathologies with similar morphological characteristics (Burger and Scheithauer, 2007).

To summarize, it is still debated whether xanthogranuloma is an entity or a culminating, reactive, secondary inflammatory process in another as yet unidentified lesion (Honegger et al., 2021). Therefore, the most appropriate therapeutical strategy is based on maximum surgical resection and, if necessary, hormone replacement therapy. Furthermore, although some reports have shown decreased residual tumor tissue using radiotherapy, its use is controversial in the management of these types of tumors (Tsai et al., 2012; Lozovanu et al., 2022; Guerrero-Pérez et al., 2023).

Finally, the prognosis of sellar xanthogranuloma is usually favorable. Since the long-term behavior of xanthogranulomatous pituitary lesions after surgical removal has not been evaluated, we recommend conducting close clinical and radiological follow-up, as has also been suggested by Yang et al. (2017), Ved et al. (2018), Shao et al. (2020), and Lozovanu et al. (2022).

By closely monitoring patients postoperatively, we can gain a better understanding of the natural history of sellar xanthogranuloma and establish appropriate non-surgical therapeutic goals.

## Conclusion

The frequent association of xanthogranulomas with other tumor lesions in the sellar region, whether or not neoplastic, suggests a reason for variations observed in the clinical-imaging spectrum of SX. At the same time, this demonstrates the histological heterogeneity of this clinical entity, presenting isolated xanthogranulomatous lesions and others associated with well-characterized tumors.

To conclude, our work provides new and valuable clinical, radiological, histopathological as well as ultrastructural information from five new cases reported in central Argentina. Added to the few previously reported cases, they contribute to obtaining a better understanding of the symptoms, pathogenesis, and development of SX, in order to achieve a more efficient and effective diagnosis and therapeutic action.

## Data availability statement

The raw data supporting the conclusions of this article will be made available by the authors, without undue reservation.

## Ethics statement

The studies involving humans were approved by the Local Ethics Committees of the corresponding medical centers: CIEIS—Clínica Universitaria “Reina Fabiola” and Sanatorio Allende of Cordoba, Argentina. All patients signed a written informed consent before surgery. This project was registered at the National Registry of Health Research (RePIS No. 3974). The studies were conducted in accordance with the local legislation and institutional requirements. The participants provided their written informed consent to participate in this study. Written informed consent was obtained from the individual(s) for the publication of any potentially identifiable images or data included in this article.

## Author contributions

SCF and MCB contributed to the conception and design of the project, sample collection, and manuscript writing. ACV and EG acquired and analyzed the imaging data. MLB, MJP, EM, RAD, CSS and EJH collected the clinical and MRI data. CAM and AAQ acquired the electronic microscopy images and described the immune cell ultrastructures. ALDP wrote and approved the final manuscript. CAM, AAQ, FNP, and ALDP reviewed the manuscript and contributed to quality control. All authors contributed to the article and approved the submitted version.

## Funding

This work was supported by the following funds granted to ALDP: Consejo Nacional de Investigaciones Científicas y Técnicas (CONICET PIP 2020-2023, grant no. 11220200102210), Secretaría de Ciencia y Tecnología, Universidad Nacional de Córdoba (SECyT-UNC 2018–2023, grant no. 33620180100675CB), and Agencia Nacional de Promoción Científica y Tecnológica—Ministerio de Ciencia y Tecnología (FONCYT-PICT 0950-2020-2024).

## Conflict of interest

The authors declare that the research was conducted in the absence of any commercial or financial relationships that could be construed as a potential conflict of interest.

## Publisher’s note

All claims expressed in this article are solely those of the authors and do not necessarily represent those of their affiliated organizations, or those of the publisher, the editors and the reviewers. Any product that may be evaluated in this article, or claim that may be made by its manufacturer, is not guaranteed or endorsed by the publisher.
